# Atrioventricular Block: An Unusual Presentation of Overactive Thyroid

**DOI:** 10.7759/cureus.35141

**Published:** 2023-02-18

**Authors:** Pradnya Brijmohan Bhattad, Zeynep Yukselen, Allen Filiberti

**Affiliations:** 1 Cardiovascular Medicine, Saint Vincent Hospital, University of Massachusetts (UMass) Chan Medical School, Worcester, USA; 2 Internal Medicine, Saint Vincent Hospital, University of Massachusetts (UMass) Chan Medical School, Worcester, USA

**Keywords:** bradyarrhythmia, overactive thyroid, primary hyperthyroidism, thyrotoxicosis, atrioventricular block

## Abstract

The clinical features of hyperthyroidism are varied, but bradyarrhythmia and atrioventricular (AV) block are typically not reported in hyperthyroid patients. We present here a case of primary hyperthyroidism with symptomatic high-grade AV block as the sole presenting feature of hyperthyroidism without any obvious precipitating factors for thyroid disease or AV block. This case highlights a rare presentation of high-grade AV block with the risk of progression to complete AV block as a complication of an untreated overactive thyroid.

## Introduction

Conduction abnormalities are rare cardiovascular complications of hyperthyroidism compared to hypertension and tachyarrhythmias, including sinus tachycardia and atrial fibrillation. PR prolongation has been reported in 2%-30% of patients with hyperthyroidism [1]. Although rare, all four types of conduction abnormalities, first-degree atrioventricular (AV) block, Mobitz type 1 (Wenckebach) and Mobitz type 2 AV block, and third-degree (complete) heart block, have been reported in Graves’ disease, thyrotoxicosis, and subclinical hyperthyroidism previously [2-5]. While some observers favor that AV block in a hyperthyroid state is seen in the presence of additional risk factors, such as infections, electrolyte abnormalities, and concomitant drug use [1,2,6], thyrotoxicosis can be the only culprit causing conduction abnormalities [7,8].

Mobitz type 2 AV block is dangerous as it may progress to complete heart block and may be associated with severe bradycardia and hemodynamic instability requiring insertion of a permanent pacemaker. We describe here a patient with symptomatic 2:1 AV block with a newly diagnosed hyperthyroid state as the sole precipitant of high-grade AV block requiring permanent pacemaker implantation.

## Case presentation

A 76-year-old female with a past medical history of diabetes mellitus type 2, hypertension, and dyslipidemia presented to the emergency room (ER) with complaints of ongoing intermittent dizziness for the preceding 2-3 weeks. She described her dizziness as a sensation of lightheadedness but denied any vertigo, tinnitus, or audiovisual changes. She reported two episodes of syncope in one week preceding the hospitalization. She sustained no injuries as a result of her syncopal events. She had no prodromal symptoms at the onset of her syncopal events other than lightheadedness. She had no symptoms of anxiety, emotional lability, weakness, tremors, palpitations, heat intolerance, increased perspiration, weight loss, or diarrhea. A complete review of systems was negative for any chest pain, shortness of breath, diaphoresis, headache, seizures, focal weakness, palpitations, cold intolerance, skin or hair changes, fevers, chills, weight or appetite changes, cough, or myalgias. She had no history of any recent immunizations, travel, infections, sick contacts, or previous exposure to radiation therapy. She had no previous history of thyroid disease. She denied any consumption of new medications or supplements. She had no history of thyroid trauma, neck procedures, recent viral infection, exposure to contrast or biotin, or herbal supplements. Her home medication included metoprolol succinate 25 mg by mouth daily for the management of hypertension, which was held on admission.

**Figure 1:**
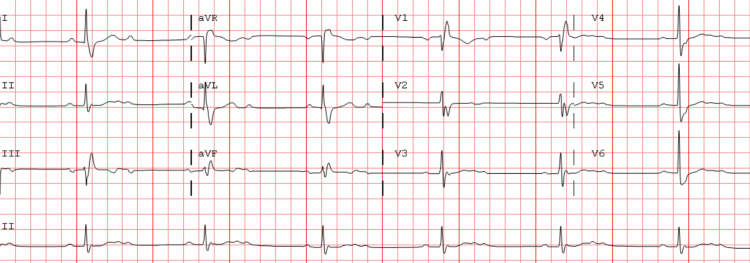
ECG showing sinus rhythm with a 2:1 AV block and a right bundle branch block ECG: electrocardiogram

Her vital signs showed blood pressure in 140s/80s mmHg, heart rate in 30s/minute, and oxygen saturation of over 95% on room air at the time of presentation. Her physical examination was unremarkable without any evidence of orthostasis. An electrocardiogram (ECG) in the ER revealed a new 2:1 AV block with a right bundle branch block (Figure 1).

She was hospitalized for further management. A complete biochemical laboratory evaluation was obtained (Table 1).

**Table 1:** Biochemical laboratory evaluation during the hospitalization TSH: thyroid-stimulating hormone, T4: free thyroxine, T3: total triiodothyronine, TSI: thyroid-stimulating immunoglobulin

Laboratory evaluation	Result	Reference
TSH level	<0.005 uU/mL	0.450-4.500 uU/mL
T4 level	1.85 ng/dL	0.7-1.7 ng/dL
T3 level	181 ng/dL	58-159 ng/dL
TSI	<0.10 IU/mL	0-0.55 IU/mL
Antithyroglobulin antibody level	<1 IU/mL	<1 IU/mL
Antithyroid peroxidase antibody titers	<8 IU/mL	0-34 IU/mL

Her serum parathyroid hormone levels, vitamin D, 25-hydroxy levels, vitamin D1, 25-dihydroxy levels, thyroglobulin, calcium levels, electrolytes, chemistry panel, and complete blood cell counts were within the normal reference range.

**Figure 2:**
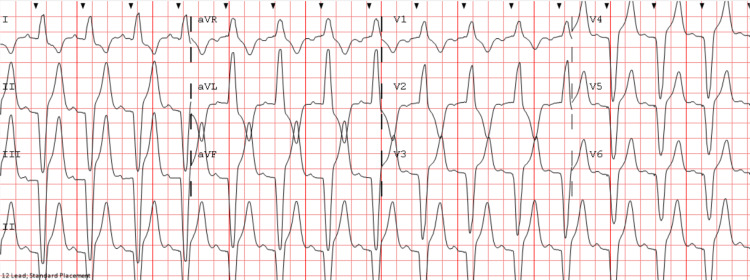
ECG in the same patient after permanent pacemaker implantation showing atrial sensed ventricular paced rhythm (PR interval of second) ECG: electrocardiogram

She was noted to have a new onset of primary hyperthyroidism with undetectable thyroid-stimulating hormone (TSH) and elevated levels of total T3 and free T4 hormones on serial testing several days apart. A transthoracic echocardiogram was normal. She was monitored off metoprolol succinate and was off any AV nodal blocking agents for over 96 hours to assess for improvement in her symptomatic high-grade AV block. Despite being completely off any AV nodal blocking agents including her home beta-blocker for over 96 hours, she continued to have significantly symptomatic high-grade AV block; hence, she underwent permanent pacemaker implantation (Figure 2).

Following well-functioning permanent pacemaker implantation, which she underwent after adequately observing her off her home metoprolol for over four days, her bradycardia resolved; however, her heart rate was found to be high normal to mildly tachycardic (90-110 bpm), and she started having increased bowel movements. Endocrinology evaluated her for symptomatic hyperthyroidism as well as undetectable repeat TSH. She was started on antithyroid treatment with oral methimazole therapy for new primary hyperthyroidism with an AV block. She will be followed as an outpatient with endocrinology along with an outpatient radioactive iodine uptake scan.

## Discussion

Early case reports suggest that AV block in a hyperthyroid state is reversible, and atrioventricular conduction returns to normal after antithyroid medications without the placement of pacemakers [1,9,10]. However, our patient was hemodynamically unstable with a heart rate in the low 30s and dizziness, requiring a permanent pacemaker along with methimazole therapy. Pacemaker requirement in hemodynamically unstable AV block in hyperthyroidism is not uncommon. In 1980, a review regarding AV block in hyperthyroidism by Miller et al. concluded that a temporary pacemaker is required in patients with Wenckebach and higher-degree AV block, and the pacemaker can be removed safely after antithyroid treatment [11]. Kuo et al. reported on a 67-year-old male patient with hyperthyroidism who presented with syncope and angina. They discharged the patient with a permanent pacemaker and methimazole treatment due to symptomatic Mobitz type 1 AV block [12]. Similarly, Ozcan et al. reported that eight out of nine patients with second-degree and/or third-degree AV block in the setting of thyroid dysfunction required permanent pacemaker placement even after normalization of thyroid status with the antithyroid treatment [13]. Therefore, regardless of the improvement of thyroid status, patients with symptomatic second-degree Mobitz type 2 AV block and those with complete AV block with or without symptoms should undergo permanent pacemaker implantation.

Several hypotheses have been suggested to explain the pathophysiology of AV block caused by a hyperthyroid state. Cardiac morphologies in autopsies of two patients with fatal hyperthyroidism showed myocardial edema, myocyte hypertrophy, myocyte necrosis, and fibrosis, which might be the cause of AV block [14]. Almange et al. suggested that a direct effect of thyroid hormone on the myocardium might be the cause of AV block according to the improvement of the conduction abnormality after the treatment of the thyrotoxicosis in their patients [15]. Singer and Shvartzman hypothesized that the most likely mechanism of second-degree AV block was an autoimmune response causing infiltration of the cardiac conduction pathways if the underlying cause of the hypertension is Graves’ disease [16]. Although the pathophysiology of overt or subclinical hyperthyroidism in the cardiovascular system is not clear yet, it is well known that a hyperthyroid status increases mortality from cardiovascular disease [17].

As discussed above, previous authors proposed that AV block may occur in the setting of electrolyte abnormalities, infectious diseases, or drug use, such as digitalis and beta-blockers [13]. Our patient did not have any obvious precipitating factors such as electrolyte abnormalities, infection, or concomitant disease suggested by previous case reports. She was on metoprolol for the management of her hypertension. However, conduction abnormality with a high-grade AV block is unlikely to be caused by a beta-blocker as it was held on admission, and the AV block did not improve after discontinuation of beta-blocker therapy; her symptoms even progressed, requiring intensive monitoring.

Drugs that slow AV conduction including digitalis, beta-blockers, and calcium channel blockers should be avoided in hyperthyroidism as they might lead to the development of third-degree AV block with Adams-Stokes attacks. This is especially important for propranolol, a commonly used drug in hyperthyroidism. Heart rate can fluctuate in a hyperthyroid state as happened in our patient who presented with bradyarrhythmia and developed tachycardia during her hospital course. It is crucial to recognize a hyperthyroid state as a possible cause in the differentials of bradyarrhythmia in addition to hypothyroidism.

## Conclusions

Conduction abnormalities are a rare but important complication of hyperthyroidism. Permanent pacemaker implantation should be considered in patients with symptomatic high-grade AV block and in individuals with third-degree block regardless of symptoms. Beta-adrenergic blockers, commonly used for symptomatic relief in hyperthyroidism, can be life-threatening in those patients with conduction abnormalities. Therefore, thyroid status should be recognized in patients with AV block, as thyroid disease may be the underlying trigger behind the development of AV block and bradyarrhythmias.
